# SiamMixer: A Lightweight and Hardware-Friendly Visual Object-Tracking Network

**DOI:** 10.3390/s22041585

**Published:** 2022-02-18

**Authors:** Li Cheng, Xuemin Zheng, Mingxin Zhao, Runjiang Dou, Shuangming Yu, Nanjian Wu, Liyuan Liu

**Affiliations:** 1 State Key Laboratory of Superlattices and Microstructures, Institute of Semiconductors, Chinese Academy of Sciences, Beijing 100083, China; chengli17@semi.ac.cn (L.C.); zxm16@semi.ac.cn (X.Z.); zhaomingxin17@semi.ac.cn (M.Z.); yushuangming@semi.ac.cn (S.Y.); nanjian@red.semi.ac.cn (N.W.); liuly@semi.ac.cn (L.L.); 2 Center of Materials Science and Optoelectronics Engineering, University of Chinese Academy of Sciences, Beijing 100049, China; 3 The Center for Excellence in Brain Science and Intelligence Technology, Chinese Academy of Sciences, Beijing 100083, China

**Keywords:** visual object-tracking, deep features, siamese network, lightweight neural network, edge computing devices

## Abstract

Siamese networks have been extensively studied in recent years. Most of the previous research focuses on improving accuracy, while merely a few recognize the necessity of reducing parameter redundancy and computation load. Even less work has been done to optimize the runtime memory cost when designing networks, making the Siamese-network-based tracker difficult to deploy on edge devices. In this paper, we present SiamMixer, a lightweight and hardware-friendly visual object-tracking network. It uses patch-by-patch inference to reduce memory use in shallow layers, where each small image region is processed individually. It merges and globally encodes feature maps in deep layers to enhance accuracy. Benefiting from these techniques, SiamMixer demonstrates a comparable accuracy to other large trackers with only 286 kB parameters and 196 kB extra memory use for feature maps. Additionally, we verify the impact of various activation functions and replace all activation functions with ReLU in SiamMixer. This reduces the cost when deploying on mobile devices.

## 1. Introduction

Visual object-tracking is a fundamental problem in computer vision, whose goal is to locate the target in subsequent video frames based on its position in the initial frame. Visual object-tracking plays an essential role in many fields such as surveillance, machine vision, and human–computer interaction [1].

Discriminative Correlation Filters (DCFs) and Siamese networks are the dominant tracking algorithm models presently. DCF emerged much earlier than Siamese network trackers. It uses cyclic moving training samples to achieve dense sampling and uses a fast Fourier transform to accelerate the learning and applying of the correlation filters. It has the advantage of high computational efficiency. However, the design of the feature descriptors requires expert intervention, and the circular sampling produces artifacts at the search boundary that can affect the tracking results. The emergence of Siamese networks provides an end-to-end solution and eliminates the tediousness of manually designing feature descriptors while exhibiting decent tracking performance.

The Siamese network tracker treats visual target tracking as a similarity learning problem. The neural network is used to learn the similarity descriptor function between the target and the search region. The Siamese network consists of two branches. The input of one branch is the initial target image, and the other one is the search region image. The Siamese network trackers perform target localization according to the similarity between the target and the search region.

The Siamese network tracker eliminates the need for complex descriptor design, uses large amounts of labeled data for training, and learns to distinguish targets from the background. Thanks to the learning and generalizing ability of neural networks, the Siamese network tracker can track targets that do not appear in the training set. The Siamese network tracker lowers the design barrier of a general-purpose target tracker with guaranteed tracking performance.

Benefiting from the compact design, promising generalization ability, and powerful performance, Siamese network trackers have been a popular research topic in recent years. Related research can be divided into three mainstreams: bounding box prediction, robust backbone network design, and online learning strategies. More specifically, SiamRPN++ [2] and OCEAN [3] use anchor or anchor-free methods to generate a precise bounding box. SiamFC++ [4] uses GoogleNet [5] instead of AlexNet [6] as the backbone network and demonstrates the impact of backbone networks on Siamese network trackers. ATOM [7] and DIMP [8] use online template updates in the Siamese network, achieving state-of-the-art performance.

Although these methods can improve tracking accuracy and robustness, they ignore computational overhead and memory footprint, therefore limiting their applications in mobile devices. Ideally, if network parameters and intermediate feature maps fit in the processor cache without data exchange with DDR, the energy efficiency would undoubtedly increase.

The backbone network contributes directly to the performance of the Siamese network. Designing efficient and lightweight neural networks for mobile devices has attracted much attention in the past few years. SqueezeNet [9] was one of the first networks to optimize the network size, proposing to reduce the network size using downsampling and 1×1 convolutional kernels. MobileNetV1 [10] and MobileNetV2 [11] introduced a new block, which used depth-separated convolution as an alternative to spatial convolutions. This further reduced the number of parameters of the network and improved its accuracy.

We propose to build lightweight target-tracking algorithms by constructing lightweight backbone networks. We start from the best practice and build the lightweight network with the basic block of MobileNetV2. Unlike other lightweight networks, we pay extra attention to the runtime memory of the network and the impact of the activation function on the network performance. We manually design the network structure and demonstrate its merits in building lightweight tracking models.

The main contributions of this paper are summarized below:

1. We propose a novel lightweight and hardware-friendly visual object-tracking model based on the Siamese tracking scheme, namely SiamMixer.
2. We design a compact backbone consisting of patch-based convolutions and mixer modules. The patch-based convolution reduces feature map memory use by processing each image patch individually. The mixer module enhances the accuracy by merging and encoding global information of feature maps.
3. We verify the activation function impact on tracking accuracy and use ReLU as a satisfying alternative for exponential-based functions, which is favorable for Single-Instruction Multiple-Data (SIMD) operations.

Extensive experimental results demonstrate that the proposed method has comparable performance with many off-the-shelf Siamese networks, while the memory footprint is significantly lower.

The structure of this paper is as follows: Section 2 reviews the Siamese-network-based trackers most relevant to our approach and the common approaches for building lightweight neural networks. Section 3.1, Section 3.2 and Section 3.3 introduce a description of the major components of the proposed network, including the convolutional layer for feature extraction, the mixer module for global encoding of the feature map, and the cross-correlation for target localization. The training setup and the loss functions design are described in Section 3.4. Section 3.5 introduces the datasets and evaluation metrics we used. Section 4.1 introduces our experimental results and compares them with the state-of-the-art algorithms. In Section 4.2, we analyze the storage overhead of SiamMixer for weights and feature maps. Section 5 concludes the paper.

## 2. Related Work

In this section, we review the visual tracker based on the Siamese network and popular methods for building lightweight networks to illustrate how our work differs from prior work.

### 2.1. Trackers Based on Siamese Network

The Siamese network tracker treats the tracking problem as a similarity discrimination problem. The basic tracking process starts with feature extraction of the target image and the search area using a neural network. The extracted feature information is then fed into the prediction head for target localization. The key to the success of the Siamese network model is to train the neural network offline using large amounts of labeled data. This allows the network to learn similarities between the target image and the search area. A well-trained Siamese network tracker maintains stable tracking even when the target undergoes complex transformations such as rotational distortion and illumination changes. A well-constructed backbone network enables the Siamese network to perform precise tracking while ensuring real-time speed, demonstrating a good balance of tracking accuracy and speed.

Because of the advantages above, trackers based on Siamese networks have been widely studied in recent years. SiamFC [12] is a pioneering work using Siamese networks for tracking tasks and has inspired a large amount of subsequent work. SiamFC proposes to use Siamese networks for feature extraction. The similarity scores between the target and the search region are calculated using cross-correlation. The location of the target is obtained from the similarity scores map. SiamFC provides a promising idea for tracking tasks, but its use of a multi-scale search scheme cannot accommodate large scale variations and aspect ratio variations.

SiamRPN [13] proposes to use a region proposal network (RPN) to estimate the target scale, avoiding the extraction of feature maps at multiple scales and achieving more accurate bounding box prediction. RPNs are widely used in object detection tasks, which use predefined anchor boxes with offsets to predict the location of targets. OCEAN further proposes the anchor-free method based on SiamRPN. Instead of using a predefined anchor box, the anchor-free method directly outputs the offset between the target and the ground truth, which improves the network accuracy and simplifies the network structure.

The backbone network directly impacts the performance of the Siamese-networks-based tracker. State-of-the-art trackers typically employ large pre-trained networks as backbone networks. SiamFC++ tests the performance of different backbone networks in the same network framework and demonstrates the impact of backbone networks on the performance of Siamese networks. SiamDW [14] analyzes the selection conditions of the backbone network in Siamese networks and presents a new residual module that allows Siamese networks to use deep networks as backbone networks.

Although the aforementioned work achieves significant performance improvements over SiamFC, its performance is entirely dependent on the generalization capability of the network. These models that are not updated online often lead to tracking failures when the appearance of the target changes significantly. ATOM, DIMP, DSiam [15] and ROAM [16] propose potential solutions for online learning of models and combine online learning with Siamese networks to achieve state-of-the-art performance. Although many techniques for model updating have been proposed, these methods usually significantly affect the speed of the network, making it impractical to meet the requirements of real-time tracking. Therefore, simply not employing online learning remains a robust and popular choice.

The subsequent work focused on improving the accuracy and robustness of the network and achieved significant improvements. However, it also brings extra computation and a large memory footprint, thus limiting its use in practical applications.

### 2.2. Lightweight Network Structure Design

Deploying neural network algorithms on edge computing platforms is a challenging task. These platforms are characterized by limited memory resources and low processor performance, thus making it impractical to deploy current state-of-the-art models and meet real-time requirements.

The work of building lightweight networks can be divided into two camps. One starts from existing high-performance networks, optimizes the network structure, compresses the network parameters, and finally makes the network meet the requirements of edge-end deployment. The representative work is deep compression [17], knowledge distillation [18], and low bit quantization [19]. These works require trade-offs between accuracy, frame rate, and the number of parameters, introducing excessive manual involvement. The others design lightweight structures directly and then combine pruning and quantization to eventually meet the requirements of edge-end deployment. Representative work is SqueezeNet and MobileNet. However, this work focuses mainly on reducing the weight parameters of the network while ignoring the memory overhead for feature maps.

Network architecture search (NAS) [20,21] is also widely adopted to build lightweight neural networks. Early NAS usually faced the problem of training a large number of neural networks from scratch, which required a significant amount of GPU resources. Subsequent NAS work narrowed the search space through manual intervention. However, limiting the search space makes the searched network structure suboptimal, and too much manual intervention also contradicts the main objective of network architecture search.

## 3. Proposed Algorithm

**Figure 1:**
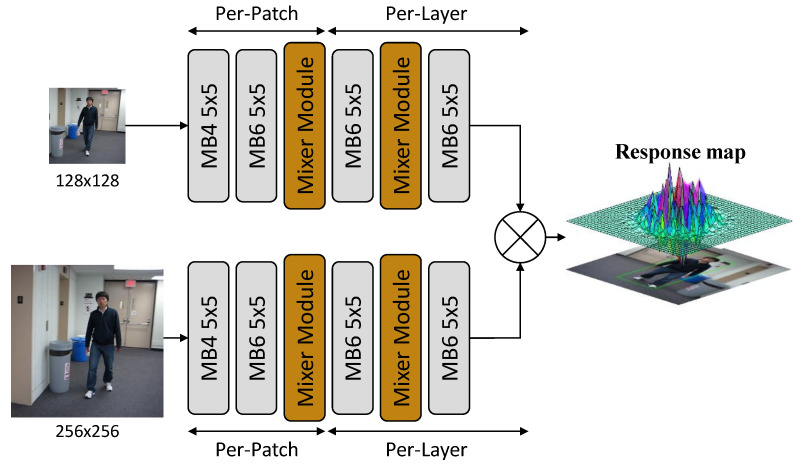
Diagram of SiamMixer network structure. The MobileNetV2 block is denoted as MB {expansion ratio} {kernel size}.

We propose to build lightweight target-tracking algorithms by constructing lightweight backbone networks, namely SiamMixer. The network can be divided into two parts, the backbone network for extracting image features and the correlation computation for object searching and locating. The diagram of the proposed tracker is shown in Figure 1.

### 3.1. Convolutional Layer

The main objective of the backbone network is to model the local and global information in the input image within a limited parameters budget. The input image is first encoded using a series of MobileNetV2 blocks. Formally, for a given input image with dimension of $C_{i}nput\times W_{i}nput\times H_{i}nput$, where $C_{i}nput$ denotes the image channels, $W_{i}nput$ denotes the image width, and $H_{i}nput$ denotes the image height.

We apply an n×n depth-wise convolutional layer followed by a pointwise (1×1) convolutional layer to conduct structural encoding. To preserve the simplicity of the network structure, the MobileNetV2 blocks used for structural encoding are implemented with the same kernel size. The architecture of the backbone network is shown in Table 1.

To reduce the runtime memory cost, we conduct the convolutional layer in a patch-by-patch order. During convolutional layer inference, one small image patch is processed at a time. Once the small image patches are processed, the memory space they occupy is freed so that the peak memory cost can be reduced. The main drawback of this method is that it is spatially constrained and unable to encode the global information of the input image. Lin [22] proposes perceptual field redistribution via NAS, thus solving the problem of constrained perceptual fields caused by patch-based inference. However, this requires an additional hyperparameter optimization in the already substantial search space. This will incur a considerable search cost.

**Figure 2:**
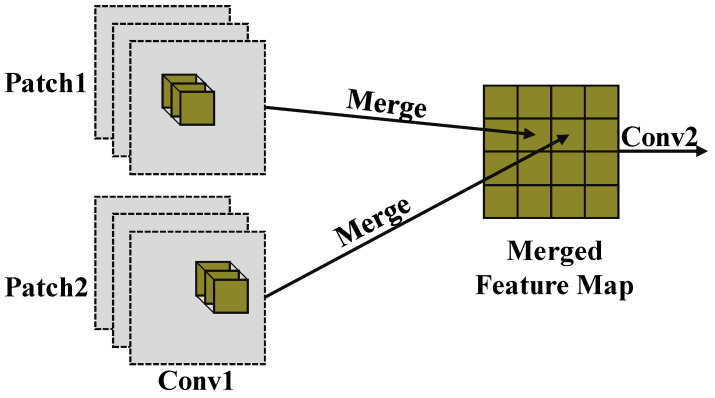
Patch-based inference is adopted to reduce the peak memory.

Therefore, we propose to use the mixer module to globally encode the convolutional feature maps. A patch-based inference example is shown in Figure 2.

### 3.2. Mixing Module

**Figure 3:**
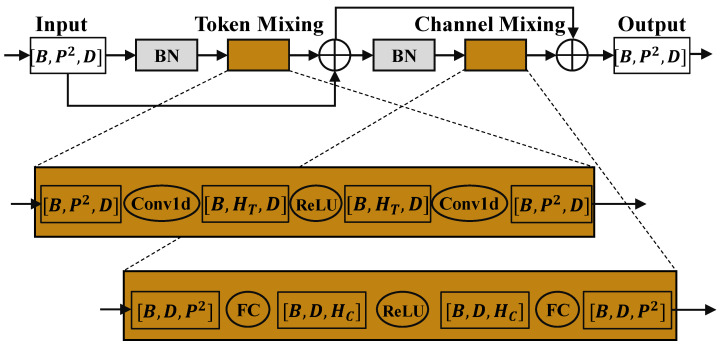
Diagram of the modified Mixer layer. Each Mixer layer contains two MLPs, one called Token Mixing MLP, and the other one called channel-mixing MLP. Token Mixing and Channel Mixing both use residual connections to ensure that the deep mixer network can be trained to converge. The input to the Mixer layer is a series of image patches that have been flattened into vectors. For each image patch, the vector dimension is $D=C\times W_{P}\times H_{P}$, where *C* is the number of channels of the patch, $W_{P}$ is the width of the patch, and $H_{P}$ is the height of the patch. The BN in the figure denotes BatchNorm (the original Mixer uses LayerNorm).

Following the convolutional layer, we use the mixer module to encode global information of the convolutional feature map. The mixer module is inspired by the network design of MLP-Mixer [23]. MLP-Mixer proposes to use a Multi-Layer Perceptron(MLP) as an alternative structure to ViT [24]. MLP-Mixer repeats the MLP-only operations on the spatial and feature channels, thus realizing the image local encoding and global encoding. A diagram of the Mixer layer is shown in Figure 3.

We combine patch-based inference with the Mixer layer to save the network from the restricted perceptual field. According to our experimental results, the combination of patch-based convolution and the Mixer layer significantly improves the accuracy of the network. To simplify the computational process, we modify the basic module of MLP-Mixer as follows:

1. Replace GELU activation function with ReLU activation function.
2. Replace LayerNorm with BatchNorm.
3. Use Conv1d for channel mixing.

Exponential arithmetic in mobile devices is usually expensive. To reduce network deployment costs, we use ReLU to replace the activation function that involves exponential operations. LayerNorms are commonly used for normalization in RNN networks because the input to RNN networks usually varies with the length of the sequence. In addition, due to the large size of the RNN network, it is not practical to use large batch size training to reduce internal covariate shift. However, LayerNorms require hidden layer statistics during both training and inference, which can slow down the inference of the networks. Since the mixer module we use has fixed dimensional inputs and the network size is small enough to use a large batch size for training, we think it is reasonable to replace the LayerNorms with BatchNorms. The original mixer network uses feature map transposition and a fully connected layer to implement channel-mixing operations. However, the transposition process would introduce unnecessary memory access and bring no computational revenue. Therefore, we use one-dimensional convolution to implement the channel-mixing process equivalently.

The adjustments we made to the network architecture made deploying the network on mobile devices easier. Experimental data shows that the impact of these adjustments on network accuracy is acceptable.

### 3.3. Target Locating

Inspired by SiamFC [12], we use the correlation operation f(z,x) to compare the template z extracted in the first frame and the enlarged search region x centered at the previous object position. If the two images contain the same object, there will be a significant response. To find the location of the target in the new image, we search pixel by pixel for the candidate location most similar to the template image. To obtain the correct scale of the target, the search area is resized into multiple scales, and the scale with the highest classification score is chosen to be the final scale. Although no high-level modeling of the target is performed, it provides a reliable and straightforward method for target localization, which is beneficial for our evaluation of the backbone network as well as for deployment on edge computing devices.

### 3.4. Training Setup

The template input and search region input sizes of SiamMixer are 128×128 and 256×256, respectively. Since the image size used for training is varied, the training images need to be cropped, resized, and padded. We train the network with positive and negative image pairings and use logistic loss and triplet loss as joint loss functions [25].

(1) $$L\left(y,v_{p_{i}},v_{n_{i}}\right)=L_{l}\left(y,v_{p_{i}},v_{n_{i}}\right)+L_{t}\left(y,v_{p_{i}},v_{n_{i}}\right)$$

where $L_{l}$ and $L_{t}$ denote logistic loss and triplet loss, respectively. $v_{p_{i}}$ is the score of the positive sample candidate. $v_{n_{i}}$ is the score of the negative sample candidate, and y∈[−1,+1], which corresponds to the ground truth.

(2) $$L_{l}\left(y,v_{p_{i}},v_{n_{i}}\right)=-\frac{1}{2MN}\sum_{i}^{M}\sum_{j}^{N}\left(log\left(1+e^{-yv_{p_{i}}}\right)\left(1+e^{-yv_{n_{j}}}\right)\right)$$

(3) $$L_{t}\left(y,v_{p_{i}},v_{n_{i}}\right)=\frac{1}{MN}\sum_{i}^{M}\sum_{j}^{N}\left(log\left(\frac{e^{-yv_{p_{i}}}}{e^{-yv_{p_{i}}}+e^{-yv_{n_{i}}}}\right)\right)$$

where *M*, *N* are the number of positive and negative samples. *y* is the ground truth label. The parameters of the network can be obtained by stochastic gradient descent:

(4) argminL(y,f(z,x;θ))

where *z*, *x* and θ is the parameters of the network, target image and search region image, respectively.

Image pairs are obtained from the annotated video dataset. Both images in the image pair contain the target. The class of the object is ignored during training. The dataset is enhanced using random horizontal flips, random rotations, and random luminance changes, where the probability of random horizontal flips is 0.5%, random rotations are from $-2^{\circ }$ to $2^{\circ }$. The center of rotation is the center of the image. The random luminance variation uses the brightness factor to jitter image brightness. The brightness factor is chosen uniformly from [0.7,1.3]

### 3.5. Datasets and Evaluation Metrics

We evaluate our tracker on the target-tracking datasets OTB100 [26] and UAV123 [27].

The OTB100 [26] dataset contains 100 challenging videos. The tracker is not reinitialized when the tracker loses its target. The dataset uses two metrics to evaluate the performance of the tracker. The precision plot indicates the percentage within a given distance threshold between the center of the predicted position and the center of the ground truth. Success plot indicates the percentage of the intersection ratio between the predicted location and ground truth within a given threshold. After obtaining the precision plot and the success plot, the score at the 20-pixel threshold is designated as the precision score, and the area under the curve of the success plot is designated as the success score.

The UAV123 dataset contains 123 challenging aerial videos. Although the data sources are different, UAV123 also evaluates the performance of the tracker using both the precision plot and the success plot.

## 4. Experiment Results

Our tracker is implemented using the PyTorch framework on a computer with an Intel Xeon Silver 4114 CPU and 4 Geforce GTX 1080 GPUs. The training is performed on GOT-10k [28] dataset. We evaluated our method on OTB100 [26] and UAV123 [27] benchmark datasets, and selected the state-of-the-art algorithms for a quantitative comparison, namely LightTrack [21], SiamFC [12], SiamRPN [13], SiamRPN++ [2], OCEAN [3], GOTURN [29], MUSTer [30], MEEM [31], STRUCK [32], TLD [33], BACF [34] and KCF [35].

### 4.1. Ablation Analysis

To demonstrate the effectiveness of the proposed method, we test the performance of different network structures.

We set the hyperparameter range according to the possible application scenarios of SiamMixer. To enable the network to run on common edge devices, the number of parameters of the network needs to be kept within 1 MB, and the total computation should be within 600 M MACs [36]. Therefore, we parametrically adjust the depth of the mixer module as a way to adjust the computational and parametric values of the network. We test four structures SiamMixer-XS, SiamMixer-S, SiamMixer-M, and SiamMixer-L, corresponding to depths 1, 2, 4, and 8, respectively. We evaluate these structures on different computing devices, and the network frame rates are shown in Table 2. The success scores of these network results on the OTB100 dataset are shown in Table 3.

Our algorithms can run at more than real-time speeds on common GPU devices while maintaining a low memory footprint. On the Nvidia Jetson Xavier development board, an edge computing device, our algorithms can run at quasi-real-time speeds.

As shown in Table 2 and Table 3, the increase in the depth of the mixer module brings limited performance improvement while significantly slowing down the network and increasing the number of parameters in the network. In addition, overly deep networks degrade network performance, which is consistent with the phenomenon described in SiamDW [14]. Therefore, we believe that SiamMixer-XS should be the optimal candidate for deployment at edge computing devices. For the performance comparison, we focused on the performance of the SiamMixer-XS.

We record the success score of different structure networks on the OTB100 [26] dataset, calculate the information density (accuracy per parameters) [37,38], and compare it with the state-of-the-art models. The comparison results are shown in Table 4.

Information density [37,38] is a metric that can effectively evaluate the efficiency of using network parameters. We want to make the most of limited storage space for edge-side deployments, so we introduce this metric in the comparison.

As can be seen from the comparison results, our SiamMixer-XS has a 6.8× smaller number of parameters than LightTrack-Mobile [21], the state-of-the-art lightweight network, and an 8.16× smaller number of parameters than SiamFC [12], which has similar performance. At the same time, our SiamMixer-XS is state-of-the-art in the metric of information density, as only a minimal amount of weight storage is required.

**Figure 4:**
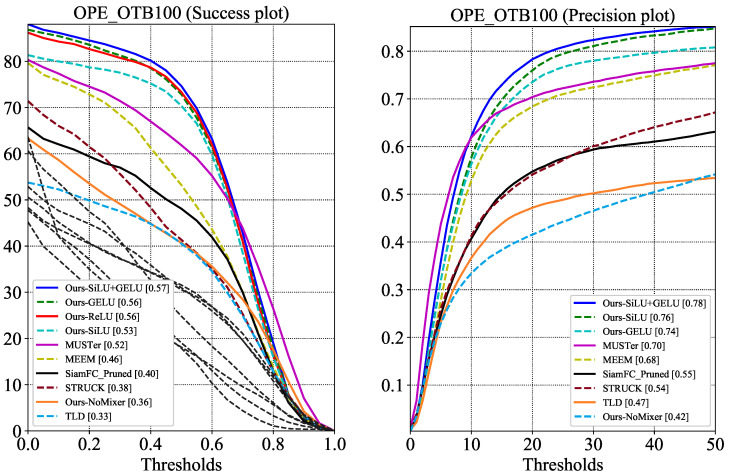
Experiment results on all OTB100 sequences. SiamFC_Pruned denotes a modified SiamFC whose memory cost is reduced to 600 kB by lowering the number of channels.

SiamMixer is built up with MobileNetV2 blocks and mixer modules. The activation functions of these modules require exponential operations, which is expensive for embedded devices. Therefore, we explore the impact of activation functions on network performance. We replace all activation functions in the SiamMixer-XS structure and test them on the OTB100 [26] dataset. In this part of the experiment, SiamFC [12] is selected as a baseline since SiamFC [12] also uses correlation for target localization. A network without a Mixer layer is also chosen to be the baseline to demonstrate the effectiveness of the combination of the per-patch convolution and the Mixer layer. The average success plot and precision plot of the trackers on the OTB100 dataset are shown in Figure 4.

Our experimental results show that when using the ReLU activation function, the overall precision score of SiamMixer-XS is 0.76, and the success score is 0.56, which is only 2.56% and 1.75% lower than the SiLU+GELU version. The loss of accuracy from replacing the activation function is acceptable but significantly reduces the hardware deployment cost, favorable for implementation with SIMD instructions.

**Figure 5:**
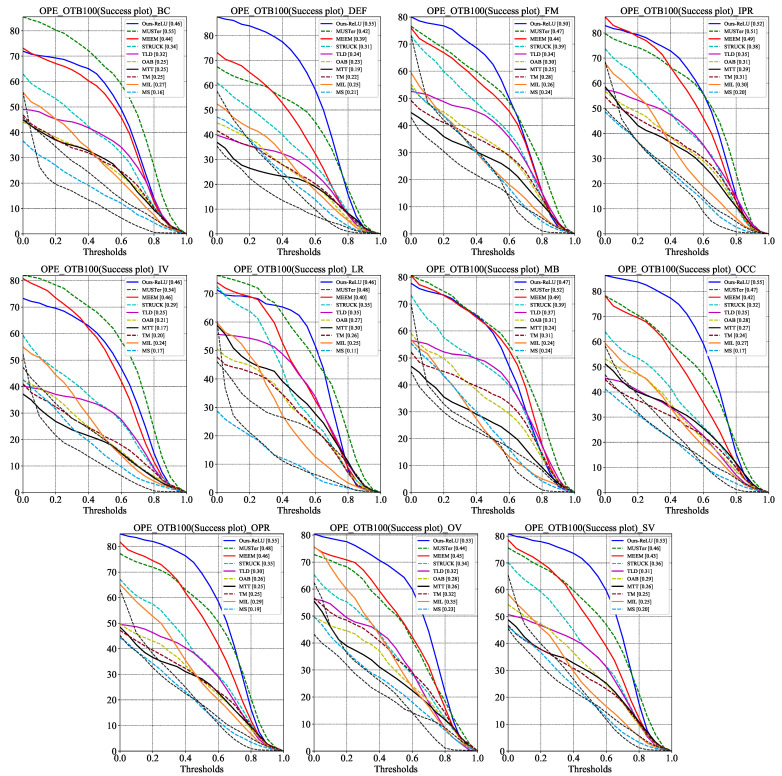
Success plots comparison on the different challenge sequences of OTB100. BC, DEF, FM, IPR, IV, LR, MB, OCC, OPR, OV, and SV denote Background Clutters, Deformation, Fast Motion, In-Plane Rotation, Illumination Variation, Low Resolution, Motion Blur, Occlusion, Out-of-Plane Rotation, and Scale Variation, respectively.

**Figure 6:**
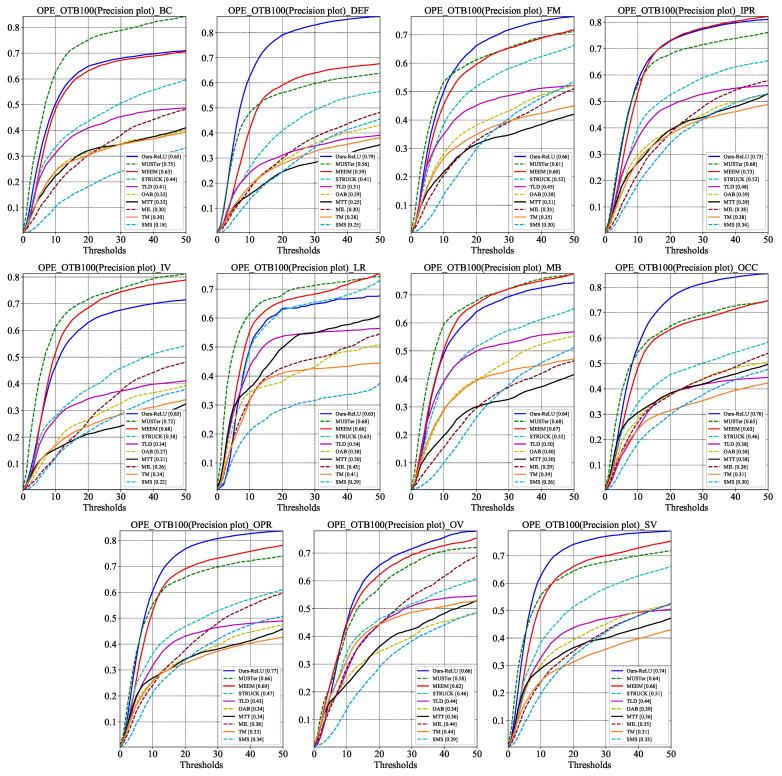
Precision plots comparison on the different challenge sequences of OTB100. BC, DEF, FM, IPR, IV, LR, MB, OCC, OPR, OV, and SV denote Background Clutters, Deformation, Fast Motion, In-Plane Rotation, Illumination Variation, Low Resolution, Motion Blur, Occlusion, Out-of-Plane Rotation, and Scale Variation, respectively.

Since different challenges in tracking place different obligations on the tracker, it is crucial to study the tracker’s tracking performance under these factors. The precision plots and success plots of SiamMixer-XS on the 11 challenge factors are shown in Figure 5 and Figure 6.

As can be seen, SiamMixer-XS achieves favorable results in most cases and, in particular, achieves significantly better results than its competitors in the DEF, FM, OCC, OPR, OV, and SV challenge.

The test results of SiamMixer-XS on the UAV123 dataset and the comparison with other algorithms are shown in Table 5. It should be noted that our model is not optimized for specific challenge scenarios, nor does it use an online learning strategy. This indicates that our network has good generalization ability, and the training process is relatively simple. Moreover, our algorithm was trained only on the GOT10k dataset and was not fine-tuned with any images from the OTB100 and UAV123 datasets. This validates the robustness and accuracy of our algorithm.

**Figure 7:**
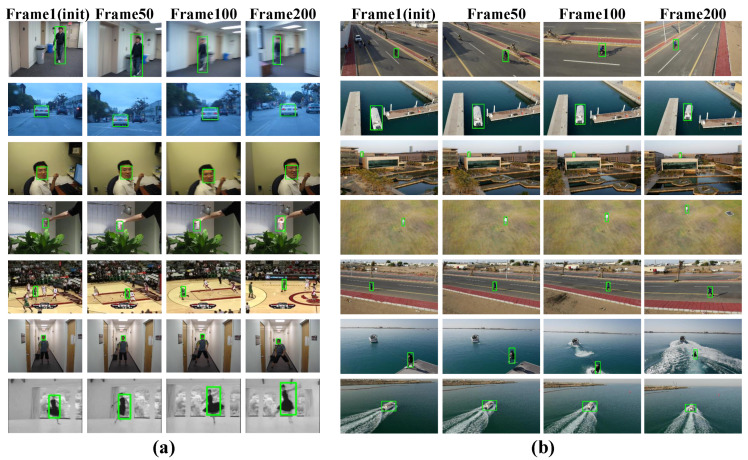
(**a**) Snapshot of the SiamMixer-XS tracking result on OTB100 [26] dataset. (**b**) Snapshot of the SiamMixer-XS tracking result on UAV123 [27] dataset.

The snapshot of the SiamMixer-XS tracking result on OTB100 [26] dataset is shown in Figure 7a. The snapshot of the SiamMixer-XS tracking result on UAV123 [27] dataset is shown in Figure 7b.

### 4.2. Storage and Analysis

Typically, algorithm analysis focuses on the computational complexity and workload while ignoring the runtime memory requirements. However, for practical application scenarios, target-tracking algorithms are usually deployed to computing devices with limited memory space and computational resources. Therefore, in addition to the computational workload and weight parameter analysis, we also analyze the storage requirements of each network layer.

**Figure 8:**
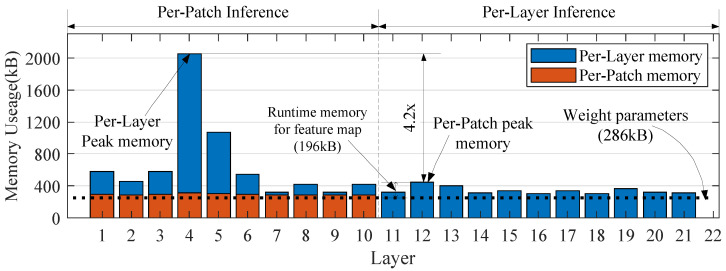
Memory use distribution of SiamMixer-XS. Using per-patch inference, we can significantly reduce the peak memory cost of the SiamMixer. Other variants of SiamMixer simply change the depth of the mixer module, and per-patch inference has the same effect on these variants.

For the convolutional layer, its weight parameters can be calculated by:

(5) $$W_{Conv}=\sum_{l=1}^{L}k_{c}^{2}\times C_{in}\times C_{out}$$

where $k_{c}$, $C_{in}$ and $C_{out}$ are the kernel size, the input channel number and the output channel number, respectively. And the feature maps storage requirement can be calculated by:

(6) $$F_{Conv}=max\left(\frac{\left(H-k_{c}\right)\times \left(W-k_{c}\right)\times C_{out}}{S}\right)$$

where *H*, *W*, and *S* are the input feature map height, width, and convolution stride, respectively. During patch-based inference, both *H* and *W* are equal to patch size. The patch size determines the perceptual field and computation load. A large patch size at the beginning of the network leads to a less effective memory reduction and overlapping perceptual fields in the later stages of the network. In SiamMixer, we set the patchsize=16 and use the mixer module to increase the network perceptual field in the latter stages of the network. Since no residual blocks are adopted during the convolution process, the feature map of the previous layer can be overwritten after the computation of one convolutional layer, so that the storage requirements can be lower. The mixer module consist of fully connected layer so its weight parameters and the feature maps storage requirement can be calculated by :

(7) $$W_{Mixer}=\sum_{l=1}^{L}D\times P^{2}\times C_{in}\times C_{out}$$

(8) $$F_{Mixer}=P^{2}\times D$$

where *D* and *P* are the hidden dim and patch number of the Mixer layer, respectively. The actual storage cost of the SiamMixer-XS is shown in Figure 8.

As shown in the figure, patch-based inference reduces its runtime memory by 4.26× and the peak extra memory for the feature map is only 196 kB, which expands the design space of the algorithm and makes it possible to deploy the algorithm on mobile devices or to work with other algorithms.

## 5. Conclusions

In this paper, a lightweight target-tracking network structure is proposed. We use a simple and efficient backbone network to extract features from the target and searching area. We use a patch-based convolutional layer to encode local features of the image. The mixer module is employed for global feature encoding. By combining the advantages of CNNs and mixer networks, our network achieves a good balance of performance, number of parameters, and runtime memory. Furthermore, we deploy this novel tracking algorithm on edge computing hardware and achieve real-time visual object-tracking. 

## Figures and Tables

**Table 1:** Architecture of backbone network. The patch-based inference layer is annotated with ${}^{☆}$.

Layer	Size	Channel in	Channel out	Stride	Expansion
MB1 ${}^{☆}$	5×5	3	16	1	4
MB2 ${}^{☆}$	5×5	16	32	1	6
Mixer1 ${}^{☆}$	-	32	32	-	-
MB3	5×5	32	64	1	6
Mixer2	-	64	128	-	-
MB4	5×5	128	256	1	6

**Table 2:** Average frame rate of 4 structural variants of SiamMixer scored on OTB100.

Name	GTX1080	TeslaV100	Jetson Xavier
SiamMixer-XS	55.89 fps	131.35 fps	26.64 fps
SiamMixer-S	49.70 fps	117.02 fps	22.92 fps
SiamMixer-M	45.71 fps	115.45 fps	22.10 fps
SiamMixer-L	23.74 fps	89.51 fps	18.46 fps

**Table 3:** Success score of 4 structural variants of SiamMixer scored on OTB100, ↑ denotes that higher value is better.

Name	SiamMixer-XS	SiamMixer-S	SiamMixer-M	SiamMixer-L
Success Score ↑	0.561	0.576	0.571	0.556

**Table 4:** Model Analysis, ↑ denotes that higher value is better, ↓ denotes that lower value is better.

Name	Par. ↓ ^1^	FLOPs. ↓	S.S. ↑ ^2^	S.P. ↑ ^3^	S.G. ↑ ^3^
Ours-XS	0.286 M	351.364 M	0.561	1.962	1.597
Ours-S	0.389 M	351.857 M	0.576	1.481	1.637
Ours-M	0.593 M	352.814 M	0.571	0.963	1.644
Ours-L	1.003 M	352.841 M	0.556	0.554	1.618
L.T.Mobile [21] ^4^	1.97 M	528.88 M	—	—	—
SiamFC [12]	2.336 M	3.179 G	0.583	0.250	0.183
SiamRPN [13]	90.436 M	25.553 G	0.637	0.007	0.025
SiamFC(VGG) [14]	9.220 M	12.221 G	0.61	0.066	0.050
SiamRPN++ [2]	23.7 M	40.89 G	0.696	0.029	0.017
OCEAN [3]	25.9 M	20.3 G	0.683	0.026	0.034
SiamFC(R.33) [14] ^5^	21.3 M	5.98 G	0.55	0.026	0.092
GOTURN [29]	114 M	0.977 G	0.45	0.004	0.461

^1^ Par. denotes the weight parameters. ^2^ S.S. denotes the success score in OTB100. ^3^ S.P. and S.G. denote for the success score per Parameter and success score per GOPs, respectively. ^4^ L.T. denotes the LightTrack [21]. ^5^ R.33 denotes the ResNet33 [39].

**Table 5:** Comparison of SiamMixer-XS with five representative trackers in UAV123 species. ↑ means the higher the score the better. S.S. denotes the success score.

Name	Ours-XS	BACF [34]	KCF [35]	SiamDW-RPN [14]	SiamFC [12]	SiamPRN [13]
S.S. ↑	0.469	0.458	0.331	0.457	0.485	0.557

## Data Availability

The publicly available benchmark datasets we use can be accessed at http://cvlab.hanyang.ac.kr/tracker_benchmark/datasets and https://cemse.kaust.edu.sa/ivul/uav123 (OTB100 is accessed on 11 December 2021, UAV is accessed on 15 December 2021).
